# Development of a Novel Tissue Blot Hybridization Chain Reaction for the Identification of Plant Viruses

**DOI:** 10.3390/plants11172325

**Published:** 2022-09-05

**Authors:** Fiona Filardo, Peter Vukovic, Murray Sharman, Cherie Gambley, Paul Campbell

**Affiliations:** Queensland Department of Agriculture and Fisheries, Ecosciences Precinct, GPO Box 267, Brisbane, QLD 4001, Australia

**Keywords:** tissue blot, hybridization chain reaction, self-assembly, plant viruses, TYLCV, polerovirus, luteoviruses, CMV, AMV

## Abstract

Assays for the high throughput screening of crops for virus monitoring need to be quick, easy, and low cost. One method involves using tissue blot immunoassays (TBIA), where plant stems are blotted onto nitrocellulose membrane and screened with available antibodies against a range of viruses. TBIAs are inexpensive but limited by antibody availability and specificity. To circumvent the antibody limitations, we developed the tissue blot hybridization chain reaction (TB-HCR). As with TBIA, plant stems are blotted onto a nitrocellulose membrane, however, TB-HCR involves using nucleic acid probes instead of antibodies. We demonstrated for the first time that TB-HCR can be used for plant viruses by designing and testing probes against species from several virus genera including *begomovirus*, *polerovirus*, *luteovirus*, *cucumovirus*, and *alfamovirus*. We also explored different hairpin reporter methods such as biotin/streptavidin-AP and the Alexa Fluor-488 Fluorophore. TB-HCR has applications for low-cost diagnostics for large sample numbers, rapid diagnostic deployment for new viruses, and can be performed as a preliminary triage assay prior to downstream applications.

## 1. Introduction

Techniques for virus identification over the last decade have improved significantly and allowed for more precise identification of viruses. However, most of these techniques such as reverse transcription (RT)-PCR, high throughput sequencing, loop-mediated isothermal amplification (LAMP), and real-time PCR are expensive when large numbers of plant samples need to be screened. Breeding programs aimed at developing virus resistant lines often test large numbers of plant samples (approx. 30,000 per year; Joop van Leur pers. comm.) for multiple viruses. Plant surveys aimed at identifying a range of viruses across a broad range of plant species and families can also involve screening hundreds of samples for multiple viruses. 

One assay for the high throughput screening of plant samples is the tissue blot immunoassay (TBIA) where the stems of each plant are blotted onto nitrocellulose membranes [1]. Membranes are then washed and processed using antibodies that either target a group of viruses or a specific virus [2]. This cheap and easy method allows for many samples to be screened at the same time and membranes can be re-processed with different antibodies or stored for longer periods of time for later analysis with no loss of sensitivity. In addition, membranes are easy to transport from overseas or isolated locations prior to processing. 

The application of TBIA is limited by the availability and specificity of antibodies. Begomoviruses are antigenically closely related, so the production of antibodies to specific species is difficult, if not impossible. The BLRV-5G4 monoclonal antibody (McAb) is commonly used to identify poleroviruses and luteoviruses, but there are limited antibodies that can distinguish between species within the families as they are antigenically related [2,3]. If antibodies are unavailable for virus identification, they can be produced via an expensive and lengthy process. Polyclonal antibody (PcAb) production may yield a useful PcAb but may produce ones that are not effective or, as is often the case with PcAbs made to poleroviruses, show cross reactivity with similar viruses. McAbs are usually selected to be more species-specific, but they are produced in limited volumes and the hybridomas need to be maintained regularly for continued supply.

An attractive alternative to antibodies are nucleic acid probes that can target either a specific virus or a group of viruses. The problem with using probes is the low signal for visualization of the virus–probe interaction on the membrane. This is due to the probe binding to one genome per virion compared to many binding sites per virion for antibodies. Probes can be directly labelled but the signal, especially with low titer viruses, would not be visible on a tissue blot assay, or requires more extensive detection regimes such as chemiluminescence. 

To improve the signal-to-background ratio for visualization of the probe targets, research has focused on in situ hybridization chain reactions (HCR) [4,5,6,7,8,9,10,11,12,13,14,15,16,17,18,19], which utilizes two kinetically trapped nucleic acid hairpin (HP) molecules (H1 and H2) that act as amplifiers. Probes are designed to the target and flanked by initiator strands. When the hairpins arrive and interact with the initiator strand on the probe, it triggers a chain reaction in which H1 and H2 sequentially nucleate and open to assemble into a long-nicked double-stranded amplification polymer. HCR amplification is used with a diverse array of output/reporter signals such as fluorescence [4,7,9,10,14,15,17], chemiluminescence [11,13], and colorimetric [11,14,16,18] detection. 

In this study, we set out to determine whether the HCR system could amplify the probe–virus interactions in the virus infected plant samples blotted onto a nitrocellulose membrane. We designed and tested probes targeted against different virus genera such as begomoviruses, poleroviruses, luteoviruses, a cucumovirus, and an alfamovirus. We also explored different hairpin and reporter methods such as biotin/streptavidin-alkaline phosphatase, biotin/anti-biotin-alkaline phosphatase, digoxigenin (DIG)/anti-DIG-alkaline phosphatase, fluorescein amidites (FAM)/anti-fluorescein-alkaline phosphatase and directly labelled hairpins with the Alexa Fluor 488 fluorophore. Using the TB-HCR assay, we were able to visually distinguish between the viral positive plant blots and negative blots and show that this assay can be used as an inexpensive triage-based assay prior to downstream applications.

## 2. Results

### 2.1. Principles of TB-HCR Method and Probe Design

#### 2.1.1. Principles of TB-HCR

**Figure 1:**
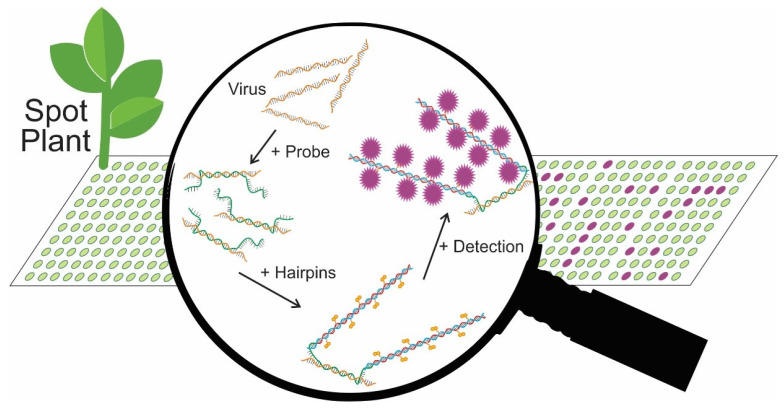
The tissue blot hybridization chain reaction (TB-HCR). Plants stems are cut and blotted onto the nitrocellulose membrane. Proteins, DNA, and RNA adhere to the membrane. DNA probes, designed to complement a specific virus or a group of viruses and contain flanking initiator sequences, attach to virus DNA/RNA on the membrane. The addition of metastable hairpins (H1 and H2) interact with the probe initiators, triggering a chain reaction in which H1 and H2 hairpins sequentially open to assemble into long double-stranded polymers. Either both or just one hairpin contains a reporter such as biotin, amplifying the signal-to-background ratio of the virus:probe interaction. Detection can then be visualized via the reporter on the hairpins.

We designed a tissue blot hybridization chain reaction assay (TB-HCR) for the detection of viruses in plants. The design of the assay is shown in Figure 1. Cross sections of each plant stem were blotted onto nitrocellulose membranes where DNA, RNA, and proteins adhere. DNA probes designed to a specific virus, or a group of viruses, hybridize to virus DNA/RNA on the blot. Following probe hybridization and washing, metastable hairpins (H1 and H2), with at least one hairpin containing a reporter such as biotin were added. The 3′ overhang and stem of the hairpins were complementary to one of the initiator sequences on the DNA probe (H1 to initiator 1 and H2 to initiator 2). The other stem of each hairpin was complementary to the opposing hairpin. The H1 hairpin binds to the initiator 1 sequence on the DNA probe, the hairpin opens and the free portion of H1 binds to a H2 hairpin, which causes the H2 hairpin to unravel, exposing the stem complementary to a H1 hairpin that binds and unravels, thus triggering a chain reaction and the assembly of long double stranded polymers. These long polymers containing, for example, biotin, amplify the signal-to-background ratio of the virus:probe interaction. Detection can then be visualized via the reporter on the hairpins. In the case of a biotin reporter, streptavidin-alkaline-phosphatase (AP) was added, followed by the addition of the substrate BCIP/NBT, causing a purple signal that can be directly visualized on the blot (Supplementary Movie S1).

#### 2.1.2. Probe and Hairpin Design

DNA probes are designed to target specific viruses or a group of viruses (Table 1). The DNA probes generally contain a 30—50 nt viral recognition site (complementary strand) flanked by two 36 nt initiators and a 4 nt spacer separating the initiator and viral sequence. DNA probes were designed to target two phloem limited viruses: tomato yellow leaf curl virus (TYLCV *Geminiviridae*, genus: *begomovirus*; probes TYLCV-P1 to P4) and the polerovirus (family *Solemoviridae)* turnip yellows virus (TuYV; probe HCR-A1). Probes for systemic viruses were designed to target the cucumovirus, cucumber mosaic virus (CMV; probes for subgroup 1—CMV-SG1-PR, CMV-SG1-PR2, CMV-SG1-PR3 and subgroup 2—CMV-SG2-PR, CMV-SG2-PR2, CMV-SG2-PR3) and the alfamovirus, alfalfa mosaic virus (AMV; probes AMV-CP-PR1 and 2, AMV-MP) from the family *Bromoviridae* (Table 1).

We also designed DNA probes or groups of probes to target all poleroviruses and luteoviruses (family *Tombusviridae*) to be used in place of the broad-spectrum TBIA BLRV-5G4 antibody [2]. The probe series HCR-A2 and -A3: TuYV-PR2 and PR3: PBMYV-PR2 and -PR3: BLRV-PR3: and SbDV-PR2 and -PR3 (Table 1) target the poleroviruses TuYV and phasey bean mild yellows virus (PBMYV) and the luteoviruses bean leaf roll virus (BLRV) and soybean dwarf virus (SbDV). These probes were designed in the polerovirus and luteovirus conserved coat protein region and are therefore not necessarily specific for the target virus but were designed to detect the group. 

The hairpins and complementary initiator sequences on the probe were as described by Choi, Beck, and Pierce [4]. Hairpins were labelled with biotin, Alexa Fluor 488, DIG, or FAM reporters (Table 1). 

### 2.2. TB-HCR for TYLCV

#### 2.2.1. Optimization of TB-HCR Procedure

**Figure 2:**
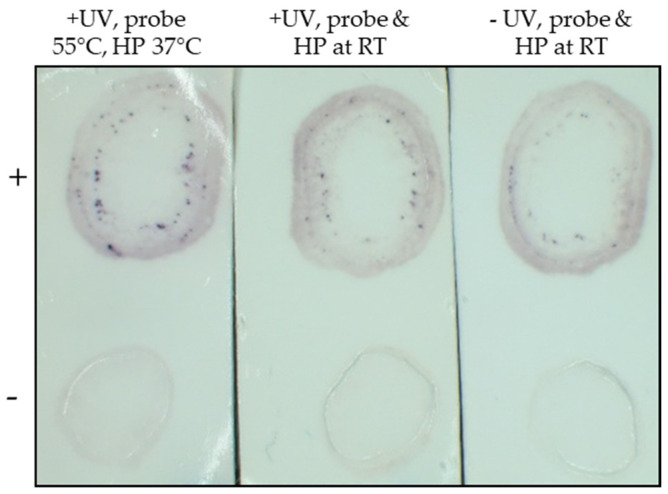
The TYLCV positive (+) and negative (−) blots were processed using the DNA probe TYLCV-P1 and the biotin labeled hairpins under varying conditions. Blots were either UV crosslinked (+UV) or not (−UV) prior to washing. Blots were probed with TYLCV-P1 at 55 °C or room temperature (RT) for 20 min and the hairpin step (HP) was carried out at either 37 °C or room temperature for 20 min, prior to staining. Deep purple spots were observed in the phloem on the TYLCV positive blots only.

To optimize the TB-HCR procedure, TYLCV positive and negative tissue blots were probed with a TYLCV probe (TYLCV-P1) and biotin labelled hairpins under various conditions. Several steps or conditions were found to improve the signal, both the intensity and number of positive spots for TYLCV on the blot. First, exposing tissue blots to UV light (1500 kJ/cm^2^) for 1 min before washing with 2x SSC improved the TYLCV positive signal (Figure 2). Second, after three washes with 2x SSC, a wash in fresh hybridization buffer before hybridizing the blot with the DNA probe was found to be crucial for assay success (data not shown). Third, the hybridization temperature was found to be optimal at 55 °C for at least 10 min for the TYLCV-P1 probe compared with either room temperature (Figure 2) or 37 °C (data not shown). 

Additionally, after probe hybridization and washing, the blot was incubated with pre-prepared hairpins—hairpins were heated at 95 °C for at least 1 min and left at room temperature to form metastable hairpins before use. We found that the hairpins should be prepared at least 30 min before use but could be pre-prepared and left at room temperature for up to 14 days with no change in activity (data not shown). The TYLCV signal improved, and the reaction was quicker when the hairpin chain reaction was carried out at 37 °C compared with room temperature (Figure 2).

#### 2.2.2. Using Multiple TYLCV Probes Improves the Signal

**Figure 3:**
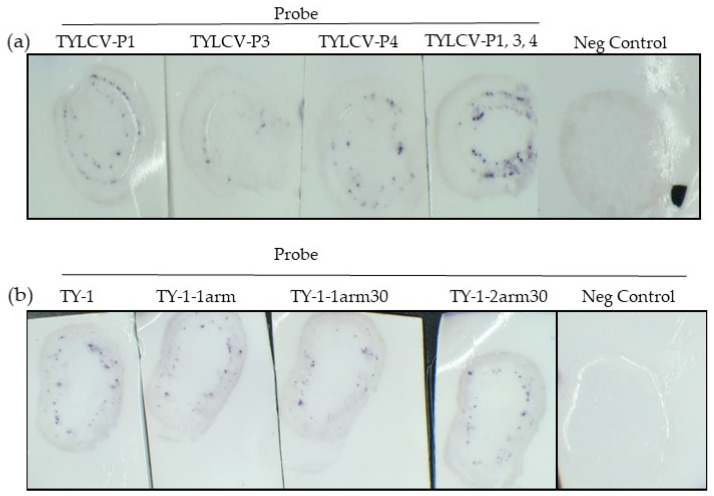
The various TYLCV probes. (**a**) TYLCV positive blots were probed with TYLCV-P1, -P3, or P4 or all three probes together, TYLCV-P1, -P3, and-P4. The negative control blot was incubated with all three probes. (**b**) Positive TYLCV blots were probed with TYLCV-P1, TY-1-1arm, TY-1-1arm30, and TY-1-2arm30. The negative control blot shown was probed with TYLCV-P1. The other negative control blots appeared the same (not shown). All blots were probed at 55 °C, followed by incubation at 37 °C with the biotin hairpins Hpin-B1-1T and Hpin-B1-2.

To improve the strength and visualization of the TYLCV signal on the blot, three more TYLCV probes targeting different regions of the genome were designed—TYLCV-P1 was positioned in the coat protein (500 nt from the start of CP), TYLCV-P2 was in the C1 gene, TYLCV-P3 was in the movement protein, and TYLCV-P4 was 700 nt into the coat protein (Table 1). All probes were able to amplify TYLCV on the blot to varying degrees. TYLCV-P2 (data not shown) and TYLCV-P3 were not as efficient at amplifying the TYLCV signal on the blot as TYLCV-P1 and P4 (Figure 3). This suggests that probes targeted to different regions of the viral genome may influence the effectiveness of the probe. 

When three probes targeting TYLCV were used together (TYLCV-P1, P3, and P4), the positive signal of TYLCV was markedly increased (Figure 3).

#### 2.2.3. Different TYLCV-P1 Probe Arrangements

Probes were designed as per Choi, Beck, and Pierce [4], with a 50 nt viral sequence flanked by two initiator sequences (Table 1). Shorter probes were also designed based on TYLCV-P1 (Table 1) to determine whether the probe length is important. The probe TY-1-1arm contains the 50 nt viral sequence but has only one initiator. TY-1-1arm30 has a 30 nt viral sequence and one initiator and TY-1-2arm30 has a 30 nt viral genome and two initiator sequences (Table 1). The results showed that a viral sequence of 30 nt and 50 nt could successfully amplify the TYLCV signal on the blot, suggesting that the probe viral sequences can range between 30 and 50 nt (Figure 3). Probes with one initiator sequence (one arm) were also able to amplify the TYLCV signal, however, the number of spots and intensity of the signal was slightly reduced (Figure 3).

### 2.3. TB-HCR with Different Viruses

#### 2.3.1. Phloem Limited Viruses

**Figure 4:**
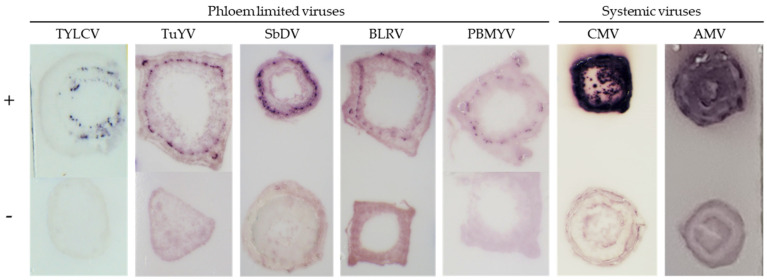
The TB-HCR targeting different viruses. Phloem limited viruses: TYLCV positive (+) blots and healthy negative control (−) blots were processed using DNA probes TYLCV-P1, TYLCV-P3 and TYLCV-P4 and the biotin labeled hairpins. Small purple spots in the phloem region were only observed on the TYLCV positive blots. Positive blots for TuYV, SbDV, BLRV, PBMYV and the healthy controls were processed using probes HCR-A1, HCR-A2, HCR-A3, TuYV-PR2, TuYV-PR3, PBMYV-PR2, PBMYV-PR3, BLRV-PR3, SbDV-PR2, SbDV-PR3, and the biotin labeled hairpins. Small deeply purple spots in the phloem region were observed only on the positive blots. Systemic viruses: positive blots for CMV and the healthy controls were processed using all CMV-SG1 and CMV-SG2 probes and the biotin labeled hairpins. Positive AMV blots and controls were probed with AMV-CP-PR1, AMV-CP-PR2, AMV-MP-PR1, and biotin labeled hairpins. For both CMV and AMV, a deep purple color across the whole blot was observed only for the positive blots. Note: Streptavidin and AP staining can faintly stain the background of some plant blots. This faint staining is not a positive reaction, positive reactions are indicated by a deep purple color.

The TB-HCR assay was tested with other low titer, phloem-limited plant viruses. The probe HCR-A1-TuYV was designed to target the polerovirus turnip yellows virus (TuYV). Using a TuYV tissue blot processed with the HCR-A1-TuYV probe and biotin HPs, TuYV was successfully detected on the blot (data not shown and Figure 4). A control probe was designed, the A1-biotin probe, which also targets TuYV but does not contain the initiator sequences. This was to confirm that the hairpins were needed to amplify the virus:probe signal. Using this probe, no virus positive spots or stains could be visualized on the TuYV positive blot (data not shown).

To improve the signal and staining of the low titer poleroviruses and luteoviruses and to capture them as a group, 10 probes were mixed and used on tissue blots for a range of viruses. TB-HCR assays using mixed probes—HCR-A1, HCR-A2, HCR-A3, TuYV-PR2, TuYV-PR3, BLRV-PR3, SbDV-PR2, SbDV-PR3, PBMYV-PR2, and PBYMV-PR3—successfully stained only the targeted polerovirus and luteovirus positive blots and did not stain the healthy controls or other viruses such as cotton bunchy top virus, AMV, or CMV (Figure 4 and data not shown).

#### 2.3.2. Systemic Viruses

To test the systemic viruses, probes targeted to CMV subgroup 1 (probes CMV-SG1_PR, CMV-SG1_PR2, and CMV-SG1_PR3) and CMV subgroup 2 (CMV-SG2_PR, CMV-SG2_PR2, and CMV-SG2_PR3) and AMV (AMV-CP-PR1, AMV-CP-PR2, and AMV-MP-PR1) (Table 1) were designed and tested. The TB-HCR assays using all of the CMV probes together or all three AMV probes on the positive and negative blots, resulted in intense positive staining over the whole blot positive for CMV or AMV, respectively (Figure 4).

### 2.4. Different Reporter Methods

**Figure 5:**
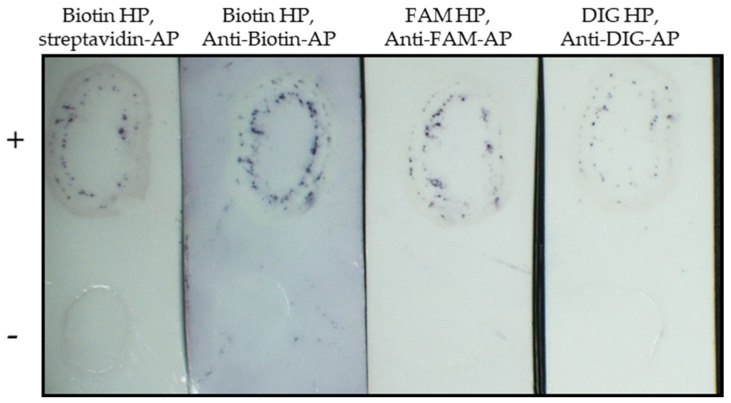
The tissue blot hybridization chain reaction (TB-HCR) with different reporter methods. TYLCV positive (+) and negative (−) blots were probed with TYLCV-P1, -P3, and -P4 and amplified and stained with: biotin HP, streptavidin-AP (used biotin hairpins, followed by incubation with streptavidin-AP, stained with BCIP/NBT): Biotin HP, anti-Biotin-AP (biotin hairpins, incubation with anti-biotin-AP, stained with BCIP/NBT): FAM HP, anti-FAM-AP (FAM hairpins, incubation with anti-fluorescein-AP, stained with BCIP/NBT): DIG HP, anti-DIG-AP (DIG hairpins, incubation with anti-digoxigenin-AP, stained with BCIP/NBT).

The TB-HCR assays described above use biotin labelled hairpins (HPs) (Table 1), followed by incubation with streptavidin-AP and stained with alkaline-phosphatase (AP) (Section 4.3). To explore other reporter methods, we used TYLCV positive blots probed with three TYLCV probes (TYLCV-P1, TYLCV-P3, TYLCV-P4) followed by amplification with (1) Biotin HPs, followed by incubation with anti-biotin-AP, and stained with BCIP/NBT (biotin HP, anti-biotin-AP), (2) FAM labelled HPs, followed by incubation with anti-fluorescein-AP and stained with BCIP/NBT (FAM HP, anti-FAM-AP), and (3) digoxigenin labelled HPs, incubated with anti-digoxigenin-AP and stained with BCIP/NBT (DIG HP, anti-DIG-AP). All reporting combinations worked to varying degrees, with the biotin HP, anti-biotin-AP combination producing the strongest staining of the positive TYLCV blot, while the DIG HP, anti-DIG-AP produced the weakest staining of the positive blot (Figure 5).

**Figure 6:**
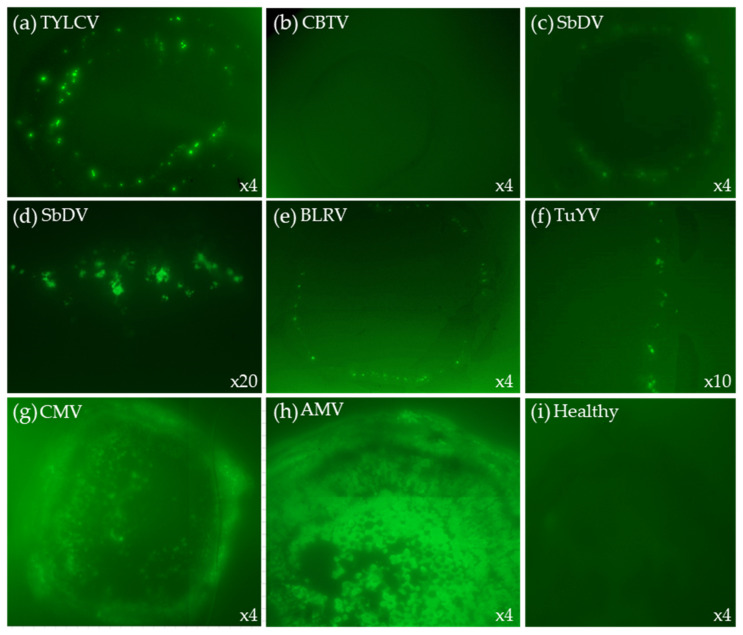
The TB-HCR using 488-HPs. The TYLCV blots were probed with TYLCV-P1, -P3, and -P4. SbDV, TuYV, BLRV, and CBTV (neg control) were probed with HCR-A1, -A2, and -A3. CMV was probed with all six CMV-SG probes and AMV was probed with two AMV-CP probes. After probing, the blots were incubated with Alexa Fluor 488 hairpins.

TB-HCR can also be used with fluorescent hairpins. We used Alexa Fluor 488 labelled HPs (488-HPs, Table 1) on a variety of different positive and negative blots. TYLCV positive and negative blots were probed with TYLCV-P1, TYLCV-P3, and TYLCV-P4, followed by HCR amplification with 488-HPs. Fluorescent green spots appeared 10—20 min after the addition of 488-HPs (incubated at 37 °C) on the TYLCV positive blots only (Figure 6) or after 25—40 min when hairpin amplification was carried out at room temperature (Supplementary Movie S2). 

Positive blots of TuYV, SbDV, BLRV, and PBMYV were probed with HCR-A1, HCR-A2, and HCR-A3 as well as negative controls of cotton bunchy top virus (CBTV) and healthy blots. After the addition of 488-HPs, small fluorescent spots were observed only on the positive blots for each of the targeted viruses (Figure 5).

For the systemic viruses, the CMV and AMV positive and negative blots were probed with all six CMV-SG probes and two AMV-CP probes, respectively. After 10—20 min of incubation with 488-HPs at 37 °C, the positive blots exhibited strong fluorescence, while the negative controls did not fluoresce (Figure 6).

### 2.5. Comparison of TBIA and TB-HCR

**Figure 7:**
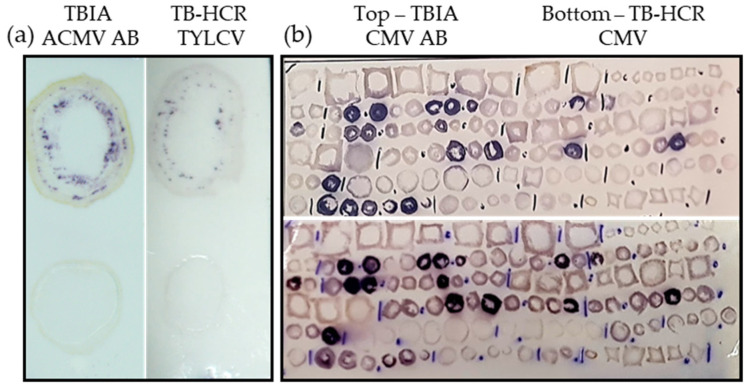
The comparison of TB-HCR with TBIA. (**a**) TYLCV positive and negative blots were processed with ACMV McAb (TBIA) or probed with TYLCV-P1, -P3, and -P4 (TB-HCR). TYLCV positive blots showed small purple spots in the phloem region of the blot. (**b**) A large, replicated survey blot was processed with anti-CMV rabbit polyclonal antibody (Agdia) or probed with the six CMV-SG probes. CMV positive plant blots stained dark purple.

To compare TB-HCR with the traditional TBIA method, which uses antibodies, positive blots of TYLCV were probed with TYLCV-P1, TYLCV-P3, and TYLCV-P4 (TB-HCR) or the broad-spectrum African cassava mosaic virus (ACMV) SCR-20 Begomovirus McAb (TBIA). CMV positive blots were compared using the six CMV-SG probes or anti-CMV rabbit polyclonal antibody. For TYLCV, both tissue blot methods successfully stained the TYLCV positive blot, although the TBIA method stained slightly stronger and showed more positive spots than TB-HCR (Figure 7a). For the systemic virus CMV, both TBIA and TB-HCR successfully stained positive CMV blots dark purple to a similar extent (Figure 7b). The blot used for the CMV test was prepared in 2015 and stored at approximately 24 °C for over 6 years, demonstrating that the target RNA remained suitable for TB-HCR on the nitrocellulose membrane for an extended period of time.

## 3. Discussion

We set out to determine whether the hybridization chain reaction assay could be applied to a tissue blot system, which traditionally uses antibodies for the detection of plant viruses. The TB-HCR assay works in detecting low titer phloem restricted viruses such as TYLCV, TuYV, SbDV, BLRV, and PBMYV and high titer viruses not restricted to phloem (CMV and AMV) in plant stems blotted onto a nitrocellulose membrane.

Begomoviruses (TYLCV) contain a circular single-stranded DNA genome in a twin (geminate) quasi-icosahedral shell. Poleroviruses (TuYV and PBMYV) and luteoviruses (SbDV and BLRV) are also encapsidated in an icosahedral shell with a monopartite linear single-stranded RNA genome. Both CMV (bromovirus) and AMV (alfamovirus) have a tripartite single stranded RNA genome forming icosahedral or bacilliform virions, respectively. We also used TB-HCR for the potyvirus bean yellow mosaic virus (BYMV), which has a single stranded RNA genome incorporated into filamentous flexible rods with its coat protein. However, to date, TB-HCR has not worked with this virus (data not shown). This could be due to the shape of the virion, and/or the accessibility of the genome for the probe to attach as the potyvirus genome is packed tightly with the coat proteins, which are interlocked. This suggests that potyvirus virions may need to be broken open prior to attaching to the nitrocellulose membrane, so that the genome is available for probe attachment. Therefore, TB-HCR is currently not optimized for all virus types and further testing is needed to find a way to break open potyvirus virions for probe attachment to RNA. Similarly to TBIA, this assay needs to be designed, tested and optimized for each target virus or virus family.

The main reporter method used in this study was the biotin hairpins, followed by streptavidin-AP and an alkaline phosphatase colorimetric stain. The advantage of this reporter/staining method is that it is cheaper than fluorescent hairpins. However, the disadvantage is that some plants contain endogenous biotin, and some purpling was observed as background staining on the blot. In most cases, the difference between background staining and a positive stain was clearly obvious. Nonetheless, in some cases, for some plants such as hibiscus, a strong staining was observed. This has also been observed with the TBIA assay. If this occurs, then another reporter system that does not use streptavidin and alkaline phosphates is advised such as the Alexa Fluor 488 hairpins. 

When comparing TB-HCR with TBIA, both methods successfully detected TYLCV positive blots, although TBIA produced a stronger more visible stain (Figure 7). TBIA uses antibodies, which bind to multiple copies of receptor sites on the outside of virions compared with TB-HCR, which relies on accessible DNA/RNA. However, one advantage of TB-HCR is that more probes can be used to increase the intensity of the stain. In the TYLCV assay, a combination of three different probes targeted to three different parts of the TYLCV genome was used. Choi, Beck, and Pierce [4] used up to 10 different probes aimed at their target. Furthermore, different hairpin arrangements that produce an increase in signal such as branching hairpins are being explored and may help to increase the signal of low titer viruses in the future [20]. 

The TB-HCR assay was designed as a triage assay that can target individual viruses or a group of viruses without the use of antibodies. When sampling large numbers of plants for a range of viruses, the TB-HCR triage assay would reduce the costs compared to PCR/RT-PCR or real-time PCR assays. We estimated that screening three hundred plants for seven different RNA viruses would cost approximately AUD 130 to AUD 140 depending on how many probes are used for the TB-HCR assay and using the biotin/streptavidin-AP method. In comparison, TBIAs would be cheaper if antibodies are available (approx. AUD 20 to AUD 35). However, RT-PCR and real-time assays are much more expensive due to the cost of the enzymes and carrying out individual PCRs for each of the seven viruses (RT-PCR is approximately AUD 5100 to AUD 5400; real-time RT-PCR is approximately AUD 4000 to AUD 5000, depending on the kits used). The main set up costs for TB-HCR are the development and synthesis of specific probes and hairpins, with fluorescent hairpins being more costly than biotin. 

The time-consuming part of both the TB-HCR and TBIA assays is the cutting, blotting, and recording of plant samples. It is advisable to make the blots in triplicate, at least. This can be conducted in the laboratory or in the field. Once the tissue blots are finished and dry, they can be easily transported and remain stable enough to be stored for many years prior to use. The TB-HCR assay only takes between 2.5 and 4 h to process, depending on whether the buffers are already set up and pre-heated. The time it takes is the same regardless of how many samples are blotted on the membrane: a blot of two or 200 plants will take the same amount of time as large numbers of plant samples can be screened at once. As with TBIA, this type of screening assay is a valuable qualitative test allowing for rapid narrowing down of the possible likely infected samples from large numbers, allowing for further testing as required. The development of a semi- or fully quantitative assay in this format is theoretically possible, but would require the further development of appropriate quantitated controls for each virus, possibly in an appropriately matched plant host. This would be the case for either TBIA or TB-HCR.

The TB-HCR assay was successfully shown to detect both low and high titer viruses from five plant virus families and is therefore likely to also work for further viruses. This novel detection method could be used as a triage assay for virus surveys, the screening of germplasm breeding lines, or the screening of imported plant lines for the presence of exotic viruses. The TB-HCR may be useful in the event of biosecurity incursions because of the short time needed to design probes and the cheap, high throughput format. The assay could also potentially be an in-field assay. Future research will focus on increasing the signal-to-background ratio and expanding the analysis to other plant viruses from different families. 

## 4. Materials and Methods

### 4.1. Plant Collection and Tissue Blots

Plant samples that were used for TB-HCR blots were collected from virus surveys or sent to us for diagnostics from various locations around Australia (Supplementary Table S1). Virus infection in plant samples was confirmed with RT-PCR or PCR. The stems of each plant were cut in cross section and blotted onto nitrocellulose membranes (Amersham™ Protran™ 0.45 NC, Germany). Tissue blots were stored in a dark place at around 24 ° C until needed.

### 4.2. DNA Probes and HCR Hairpins 

DNA probes contain one or two 36 nt initiator(s), a 4 nt spacer separating the initiator and viral sequence, and a 50–30 nt viral recognition site (Table 1). Probes were ordered from Macrogen Inc. (Seoul, South Korea). For the directly labeled A1-biotin control probe ordered from Bioneer Pacific, the 3′ end initiator sequence was removed, and the 5′ initiator sequence was made shorter to enable direct biotin attachment that would not be difficult to synthesize or become unstable. Probes were resuspended in low EDTA TE buffer pH 8.0 (10 mM Tris-HCL, 0.10 mM EDTA) to make a 10 µM working stock.

Hairpin sequences used in this study were as per Choi, Beck, and Pierce [4], DNA HCR B1; H1 and H2 (Table 1). Reporters attached to the hairpins were as follows: Biotin, Alexa Fluor 488, FAM, and DIG. Hairpins were ordered from either Macrogen Inc. (South Korea) or Integrated DNA Technologies (IDT). Hairpins were resuspended to 100 µM stocks with low EDTA TE buffer pH 8.0.

### 4.3. Tissue Blot–Hybridization Chain Reaction

Prior to washing, tissue blots were UV-crosslinked in a HL-2000 hybriLinker at 1500 kJ/cm^2^ for 1 min to aid in DNA/RNA hybridizing to the membrane. Membranes were rinsed three times (5 min per wash) in 2x SSC + 1.0% SDS at room temperature (RT). Membranes were then transferred to pre-heated (55—60 °C) hybridization buffer for 2–5 min with gentle shaking. Three different hybridization buffers were used—ULTRAhyb™ Ultrasensitive Hybridization Buffer (Invitrogen, Waltham, MA, USA), Rapid-hyb buffer (Amersham, UK) and PerfectHyb buffer (Sigma, St. Louis, MO, USA)—and all worked equally well. Membranes were transferred to fresh pre-warmed (55 °C–60 °C) hybridization buffer containing probe(s) at a final concentration of 0.05 µM (each probe) and incubated at 55 °C–60 °C for a minimum of 10 min with gentle shaking. Excess probe(s) were removed by washing once with 0.5x SSC + 0.1% SDS at 55 °C, then twice with 0.2x SSC + 0.1% SDS at 37 °C for 2–5 min each. Membranes were then washed for 1–2 min in pre-warmed (37 °C) amplification buffer (5x SSC, 0.1% Tween 20, 0.01% dextran sulfate) then transferred to fresh pre-warmed (37 °C) amplification buffer, where pre-prepared hairpins were added to start the HCR reaction. Prior to the HCR step, each hairpin was prepared separately in 5x SSC at a final concentration of 30 µM, heated at 95 °C for 90 s, then cooled to room temp for at least 30 min prior to use. The HCR step took 10–20 min at 37 °C. 

When fluorescently labeled hairpins (Alexa fluor 488) were used, fluorescence on positive blots were observed after 10–20 min when the HP reaction was carried out at 37 °C with gentle shaking or 25–40 min when incubated at room temperature. Membranes were then washed in fresh amplification buffer to wash off excess hairpins and were visualized and photographed using a Nikon i80 biological compound microscope with a FITC (465/495 nm) fluorescent filter or a Leica stereo microscope with a GFP filter. Blots were stored in a dark dry place. 

When biotin, FAM, or DIG hairpins were used for colorimetric assays, membranes were transferred and washed twice with amplification buffer + 3% BSA (bovine serum albumin; Bovogen Biologicals, Keilor East, VIC, Australia) at RT. For biotin hairpins, membranes were then transferred to fresh amplification buffer +3% BSA + streptavidin-AP (~0.4 µL in 5 mL of buffer: Invitrogen, Cat# 434322, USA) for 10 min at RT with shaking. Or alternatively, biotin, FAM, or DIG hairpins were detected with antibodies. In this case, membranes were incubated in fresh amplification buffer +3% BSA and their respective antibody; anti-biotin-AP (SIGMA, USA), anti-fluorescein-AP (Roche, Mannheim, Germany), or anti-digoxigenin-AP (Roche, Germany), respectively, for 10–30 min at RT with gentle shaking. 

Following the addition of streptavidin-AP or anti-antibody-AP, membranes were washed twice in substrate buffer (10 mM diethanolamine, 750 mM NaCl, 500 mM MgCl_2_·6H_2_O, 0.2% Tween 20, 0.01% dextran sulfate), then transferred to fresh substrate buffer containing solutions A and B from the AP conjugate substrate kit (Bio-RAD, Hercules, CA, USA) at a concentration of 5 µL/mL. Membranes were left to develop for ~10–30 min at RT until color development was observed with a Leica dissecting microscope. 

### 4.4. Tissue Blot Immunoassay

Tissue blot immunoassays (TBIA) were carried out as per Makkouk and Comeau [1]. Membranes were incubated first with an antigen-specific primary antibody, then with an alkaline-phosphatase conjugated anti-mouse or anti-rabbit antibody (Sigma catalogue numbers A3562 and A3687) followed by an alkaline phosphatase substrate (Bio-RAD, USA). Primary antibodies used were anti-CMV rabbit polyclonal antibody (Cat#: CAB 44501, Agdia, Elkhart, IN, USA) for the detection of CMV, anti-AMV rabbit polyclonal antibody (Cat#: CAB 87601, Agdia) for AMV, ACMV SCR-20 begomovirus McAb [21] for TYLCV, and the broad-spectrum monoclonal antibody BLRV 5G4 [2,3] for luteoviruses and poleroviruses.

## Figures and Tables

**Table 1:** The DNA probes and hairpins used in this study for the detection of plant viruses. Bold indicates the viral probe sequence and the underlined sequence on the hairpins is complementary to the initiator sequence on the probes.

Name	Virus DNA Probe Sequence	Probe Start (nt Position) on Reference Genome
Basic probe design	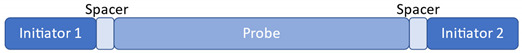	
Initiator1(init1)	GAGGAGGGCAGCAAACGGGAAGAGTCTTCCTTTACG	
Initiator2(init2)	GCATTCTTTCTTGAGGAGGGCAGCAAACGGGAAGAG	
A1-Biotin ^+^	biotin–GAGGAGGGCTCCTTTACG TATT**CTTGAGYATTCCATTAGMGAATGCYGGGCAGTCTGATAGACTCGGCCCGA**	NC_003743 (3783 nt)
AMV-CP-PR1	init1 TATT **ACACGCAGCCCGTCTGTGGCAGTATAGTATTCGTCGGTT** ATAT init2	NC_ 002025 (1355 nt)
AMV-CP-PR2	init1 TATT **TTCTCTCGACCCAAACTTCGTTGAATCGGTATGAGGGA** ATAT init2	NC_ 002025 (1775 nt)
AMV-MP-PR1	init1 TATT **ACTTCATCAGCTAGTAACATTTCCTCAGCATAAGACACT** ATAT init2	NC_ 002025 (338 nt)
BLRV_PR3	init1 TATT **ATCCTGAATTGGTCCTCTGAAGTGTCGTGCCATGCCTGTCCATTGATA** ATAT init2	NC_003369 (3601 nt)
CMV-SG1_PR	init1 TATT **TCGGCAAAGGATTAACTCGAATTTGAATGCGCGAAACAAGYTTCTTAT** ATAT init2	NC_001440 (1602 nt)
CMV-SG1_PR2	init1 TATT **ACCAGTACCGGTGAGGCTCCGTCCGCRAACATAGCAGAGATGGCGG** ATAT init2	NC_001440 (1711 nt)
CMV-SG1_PR3	init1 TATT **AACGTCTTRTTRAGTCGCGAAAGYTGYTGCGACARGACTCTAAAG** ATAT init2	NC_001440 (1393 nt)
CMV-SG2_PR	init1 TATT **CGACGGAAAGATCGGATGATGAAGGYACTTTCCGAACTGTAACCC** ATAT init2	AJ585519 (1598 nt)
CMV-SG2_PR2	init1 TATT **TAGGAATTCGTTGATGCTCGACGTCGACATGAAGTACAATCTCGTCCT** ATAT init2	AJ585519 (1820 nt)
CMV-SG2_PR3	init1 TATT **GCATCCGCACCAGAAGCGGACCGAGAACCTCTACGCGGGCGACGA** ATAT init2	AJ585519 (1278 nt)
HCR-A1-TuYV	init1 TATT **CTTGAGYATTCCATTAGMGAATGCYGGGCAGTCTGATAGACTCGGCCCGA** ATAT init2	NC_003743 (3783 nt)
HCR-A2 *	init1 TATT **CTTGAGTATKCCATCWGMGARYGGCTTGCAYTCTGATARAGACGGCCCGA** ATAT init2	NC_003056 (3328 nt)
HCR-A3 *	init1 TATT **ATATTCATGGTAGGCCTTGAGTATTCCAGAACTGAAYTCWGGCTTCTCTG** ATAT init2	NC_028793 (4017 nt)
PBMYV-PR2	init1 TATT **AGGGAGCTATATTTGCAATGGGGGTCCAGCTCATAAGCGATGGAACC** ATAT init2	MK955806 (4045 nt)
PBMYV-PR3	init1 TATT **GCCCCGTTTCCTTTGTATAGGATTCGGAACTGATCCTCGGATGCGTCG** ATAT init2	NC_028793 (4250 nt)
SbDV-PR2	init1 TATT **TCATAAGCGATGGARCCTGASGAGGTGGAAGAGGCCTCGGTGATG** ATAT init2	NC_003056 (3414 nt)
SbDV-PR3	init1 TATT **CCTCCTCTATTATTTCTCCTTCGTCGTCGTTGTCGTCCTCTGCGTTGTG** ATAT init2	NC_003056 (3189 nt)
TuYV-PR2	init1 TATT **GAAAGGGAGTTGAGTTTACAGTGTGGGTCCAGCTCGTAAGCGATGGAA** ATAT init2	NC_003743 (3901 nt)
TuYV-PR3	init1 TATT **TTGCCTTTGTAGAGGATCCTGAATTGGTCCTCGGCAACGTCGTGCCAT** ATAT init2	NC_003743 (4024 nt)
TY1-1arm	init1 ATAT **GTACTGGGCTCATTATCGAACATATTAAAAACCTGTCCAAAATCCATT**	GU178814 (807 nt)
TY1-1arm30	init1 ATAT **GCTCATTATCGAACATATTAAAAACCTGTCC**	GU178814 (800 nt)
TY1-2arm30	init1 ATAT **GCTCATTATCGAACATATTAAAAACCTGTCC** ATAT init2	GU178814 (800 nt)
TYLCV-P1	init1 ATAT **GTACTGGGCTCATTATCGAACATATTAAAAACCTGTCCAAAATCCATT** ATAT init2	GU178814 (807 nt)
TYLCV-P2	init1 ATAT **CCCAAACAGGTCAGCACATTTCCATCCGAACATTCAGGCAGCTAAGAG** ATAT init2	GU178814 (2382 nt)
TYLCV-P3	init1 ATAT **TGCAAATATTTAATAGCTAACATACAACGAAATCCGTGAACAGATTCAGG** ATAT init2	GU178814 (224 nt)
TYLCV-P4	init1 ATAT **GCCATATACAATAACAAGGCGTTTTCAGTATGGTTCTCATACTTGGCTGC** ATAT init2	GU178814 (1008 nt)
**Name**	**Hairpin Sequences**	
Hpin-B1-1T (Choi et al. 2014)	CGTAAAGGAAGACTCTTCCCGTTTGCTGCCCTCCTCGCATTCTTTCTTGAGGAGGGCAGCAAACGGGAAGAG	
Hpin-B1-2 (Choi et al. 2014)	/5′–Biotin-C6/GAGGAGGGCAGCAAACGGGAAGAGTCTTCCTTTACGCTCTTCCCGTTTGCTGCCCTCCTCAAG AAAGAATGC	
HCR B1 H1 488	CGTAAAGGAAGACTCTTCCCGTTTGCTGCCCTCCTCGCATTCTTTCTTGAGGAGGGCAGCAAACGGGAAGAG/iSp9/3AlexaF488N/	
HCR B1 H2 488	/5ATTO488N/iSp18/GAGGAGGGCAGCAAACGGGAAGAGTCTTCCTTTACGCTCTTCCCGTTTGCTGCCCTCCTCAAGAAAGAATGC	
Hpin B1-2D	DIG-GAGGAGGGCAGCAAACGGGAAGAGTCTTCCTTTACGCTCTTCCCGTTTGCTGCCCTCCTCAAGAAAGAATGC	
Hpin B1 H2-FAM	FAM-GAGGAGGGCAGCAAACGGGAAGAGTCTTCCTTTACGCTCTTCCCGTTTGCTGCCCTCCTCAAGAAAGAATGC	

^+^ A1-biotin probe targets TYLCV. * HCR-A2 and HCR-A3 probes were designed in the conserved coat protein region to target poleroviruses and luteoviruses, respectively.

## Data Availability

Data sharing not applicable.

## Supplementary Materials

The following are available online at https://www.mdpi.com/article/10.3390/plants11172325/s1, Table S1: Virus isolates used on tissue blots for TB-HCR testing. S1: The TB-HCR step-by-step protocol. Movie S1: Video concept of TB-HCR. Movie S2: Video TB-HCR with the Alexa Fluor 488 hairpins.
