# Supplementary material for: Development of a Novel Tissue Blot Hybridization Chain Reaction for the Identification of Plant Viruses
Source: Plants (Basel). 2022 Sep 5;11(17):2325. doi: 10.3390/plants11172325 (PMC9459701; doi:10.3390/plants11172325)
Supplement: Supplementary file 1 [file plants-11-02325-s001.zip › S1_TB_HCR virus isolate table.pdf]

Supplementary figures

Table S1. Virus isolates used on tissue blots for TB-HCR testing.

Virus species	Virus Genus	Host species (common name)	Isolate name
AMV	*Alfamovirus*	*Cicer arietinum*	20140930ms-1
AMV	*Alfamovirus*	*Cicer arietinum*	5223
BLRV	*Luteovirus*	*Cicer arietinum*	20140930ms-19
BLRV	*Luteovirus*	*Cicer arietinum*	20140930ms-95
BLRV	*Luteovirus*	*Vicia faba*	20150720ms-19
BLRV	*Luteovirus*	*Vicia faba*	20171013ff-148
BLRV	*Luteovirus*	*Vicia faba*	20140704ms-58
CBTV	*Polerovirus*	*Althaea officinalis*	20140930ms-115
CMV	*Cucumovirus*	*Cicer arietinum*	5638
PBMYV	*Polerovirus*	*Cicer arietinum*	20140930ms-13
PBMYV	*Polerovirus*	*Pisum sativum*	20140930ms-166
PBMYV	*Polerovirus*	*Vicia faba*	20150720ms-7
PBMYV	*Polerovirus*	*Vicia faba*	20140704ms-38
PBMYV	*Polerovirus*	*Vicia sativa*	20140903jvl-49
SbDV	*Luteovirus*	*Cicer arietinum*	20171009ff-149
SbDV	*Luteovirus*	*Cicer arietinum*	20171013ff-2
SbDV	*Luteovirus*	*Cicer arietinum*	20171013ff-57
SbDV	*Luteovirus*	*Cicer arietinum*	20171009ff-126
SbDV	*Luteovirus*	*Cicer arietinum*	20140930ms-223
SbDV	*Luteovirus*	*Cicer arietinum*	20140930ms-92
SbDV	*Luteovirus*	*Cicer arietinum*	20140930ms-223
SbDV	*Luteovirus*	*Cicer arietinum*	20151006ms-111
SbDV	*Luteovirus*	*Cicer arietinum*	20151006ms-153
SbDV	*Luteovirus*	*Cicer arietinum*	20151006ms-188
SbDV	*Luteovirus*	*Cicer arietinum*	20151006ms-290
SbDV	*Luteovirus*	*Cicer arietinum*	5436
SbDV	*Luteovirus*	*Lens culinaris*	5435
TuYV	*Polerovirus*	*Brassica napus*	20190823ff-1
TuYV	*Polerovirus*	*Brassica napus*	20190823ff-2
TuYV	*Polerovirus*	*Brassica napus*	20190823ff-6
TuYV	*Polerovirus*	*Brassica napus*	20190823ff-11
TuYV	*Polerovirus*	*Brassica napus*	20190823ff-13
TuYV	*Polerovirus*	*Brassica napus*	20190823ff-19
TuYV	*Polerovirus*	*Brassica napus*	20190823ff-24
TuYV	*Polerovirus*	*Brassica napus*	20190823ff-27
TuYV	*Polerovirus*	*Brassica napus*	20140930ms-75
TuYV	*Polerovirus*	*Brassica napus*	20140731ms-83
TuYV	*Polerovirus*	*Cicer arietinum*	20140930ms-118
TuYV	*Polerovirus*	*Cicer arietinum*	20151006ms-1
TuYV	*Polerovirus*	*Cicer arietinum*	20151006ms-3
TuYV	*Polerovirus*	*Cicer arietinum*	20151006ms-4
TuYV	*Polerovirus*	*Cicer arietinum*	20151006ms-39
TuYV	*Polerovirus*	*Cicer arietinum*	20151006ms-459
TuYV	*Polerovirus*	*Diplotaxis tenufoila*	20190823ff-30
TuYV	*Polerovirus*	*Lens culinaris*	20140930ms-139
TYLCV	*Begomovirus*	*Solanum lycopersicum*	5649
TYLCV	*Begomovirus*	*Solanum lycopersicum*	20200811P6B1-2
TYLCV	*Begomovirus*	*Solanum lycopersicum*	20200811P2B2-3
TYLCV	*Begomovirus*	*Solanum lycopersicum*	20200811P2B2-5
-	-	*Solanum lycopersicum*	Healthy tomato
-	-	*Solanum lycopersicum*	20200811P6B1-1
-	-	*Vicia faba*	20150720ms-9
		Vicia faba	20150720ms-22
