# Supplementary material for: Development of a Novel Tissue Blot Hybridization Chain Reaction for the Identification of Plant Viruses
Source: Plants (Basel). 2022 Sep 5;11(17):2325. doi: 10.3390/plants11172325 (PMC9459701; doi:10.3390/plants11172325)
Supplement: Supplementary file 1 [file plants-11-02325-s001.zip › S2_TB HCR Protocol.pdf]

TB-Hybridization chain reaction (TB-HCR)

Method developed and optimised by Paul Campbell, Fiona Filardo and Peter Vukovic.

See attached recipes for listed buffers.

Tissue printing

- Blot plant stems onto nitrocellulose membrane (Amersham Protran 0.45 uM supplied by Merk, Cat # GE10600018).
- UV-crosslink membrane in HL-2000 hybrilinker at 1500kj/cm^2 for 1 min – aids in DNA/RNA hybridizing to membrane

Note:- hairpins can be prepared at any stage (see process below) and buffers should be prewarmed

Probe hybridization stage

- Rinse membrane 3 times in 2 x SSC + 1.0 % SDS at room temperature (RT) for ≥5 mins each.
- Prepare Hybridisation (Hyb) Buffers (2 separate lots) - enough to cover membrane by at least 2mm (e.g. ~ 3mL for membrane 5 x 2 cm in small container) and pre heat to 55°C - 60 ^{o}C (temperature depends on the probe).
- Wash membrane in pre-heated Hyb Buffer and incubate at 55 ^{o}C - 60 ^{o}C for ≥5 mins.
- Prepare Probe: Add 5-8 uL of each 10uM probe (diluted in TE-low EDTA) per 1 mL **fresh** warmed Hyb Buffer (eg 3 mL pre-heated hyb buffer + 15 uL of each probe).
- Transfer membrane to Fresh Hyb buffer containing probe.
- Incubate membrane at 55°C - 60 ^{o}C for 10-30 min with gentle shaking.
- Remove excess probes by washing 1x with 0.5 x SSC + 0.1% SDS at 55°C, then 2x with 0.2 x SSC + 0.1 % SDS at 37°C for ≥5 mins each.

Amplification stage

- Prepare each labelled hairpin <u>separately</u>: Add 3 µL HP (100UM) to 10 uL of 5 x SSC. Heat HP at 95°C for 90 seconds then allow to come to room temp for ~30 min.
- Transfer membrane from wash buffer to pre-warmed (37°C) amplification buffer, enough to cover blots (~3 mL).
- When HPs are ready, transfer membrane to **fresh** pre-warmed amplification buffer (3 mL) and add each of the hairpin solutions (total 13 uL per HP).
- Incubate at 37°C with gentle shaking for 10 min - 1 hr.

Detection stage – for biotin labelled HP's

- Transfer membrane and wash twice with detection buffer + 3% BSA at RT.
- Transfer membrane to fresh detection buffer + 3% BSA + streptavidin-AP (~1:5,000 – 10,000 dilution for new Invitrogen bottle). Ie. 5 mL buffer + 120 mg BSA + ~0.4ul strep-AP.
- Incubate with shaking for 10 min at RT.
- Transfer membrane and wash 2x with RT substrate buffer for ≥5 mins each.
- Transfer membrane to fresh substrate buffer + substrate solutions A & B (5 uL / mL Substrate Buffer e.g. 25 uL each in 5 mL buffer) and leave for ~ 10 - 30 min at RT watching colour development.

Detection stage – for fluorophore HP's

- Add HP and watch for fluorescence after 10 min – 1 hr.

SOLUTIONS

**<u>20X SSC, 1L</u>**<br>175.3 g NaCl (3.0 M NaCl)<br>88.2 g Na citrate (0.3 M Na citrate; $C_6H_5O_7Na_3$)<br>+ HCL to pH 7	**<u>Amplification buffer</u>**<br>100 mL of 5 X SSC<br>100 ul Tween 20 (final is 0.1 % Tween)<br>10 mg Dextran Sulphate (final is 0.01 %)
**<u>2 x SSC + 1 % SDS, 1L</u>**<br>100 mL of 20 x SSC<br>100 ml 10 % SDS<br>Make up to 1L with ddH20	**<u>Detection Buffer – pH7.5</u>**<br>100 mM Tris-HCL<br>100 mM NaCL<br>2mM $MgCl_2$<br>0.05 % Triton X-100<br>3% BSA at time of use.
**<u>5 x SSC, 200 ml</u>**<br>50 mL of 20 x SSC<br>150 mL with ddH20	**<u>1L Detection Buffer</u>**<br>15.76 g Tris-HCL<br>5.884 g NaCL<br>0.4066 g $MgCl_2.6H_2O$<br>pH to 7.5 with NaOH<br>500 ul Triton X-100<br>3% BSA at time of use.
**<u>0.5 x SSC + 0.1% SDS, 400 ml</u>**<br>10 ml of 20 x SSC<br>4ml 10 % SDS<br>Make up to 400 ml with ddH2O	**TE buffer with low EDTA (for diluting probes)**<br>**<u>10 mM Tris-HCL pH 8.0 & 0.1 mM EDTA – 50ml</u>**<br>500 ul 1M Tris pH 8.0<br>20ul EDTA pH 8.4. 250 mM<br>49.48 ml H_2O
**<u>Substrate buffer 1L</u>**<br>NaCl – 5.84 g<br>100 mM Tris – 12.12 g<br>5mM $MgCL_2.6H^2O$ - 1.02g. pH to 9.5	**<u>0.2 x SSC + 0.1% SDS, 400 mL</u>**<br>4 ml of 20 x SSC<br>400 ul of 10 % SDS

Hybridization Buffers:
ULTRAhyb™ Ultrasensitive Hybridization Buffer (Invitrogen, Cat#: AM8670)

Rapid-hyb buffer Amersham, (Cat#: RPN1635/6)

PerfectHyb – Sigma, (Cat#: H7033) – can get in 1L cheaper

Streptaviden AP – Invitrogen (Cat#: 434322)

AP conjugate substrate kit – Bio-Rad (Cat#: 1706432)
